# Supplementary material for: Endosperm Evolution by Duplicated and Neofunctionalized Type I MADS-Box Transcription Factors
Source: Mol Biol Evol. 2021 Dec 13;39(1):msab355. doi: 10.1093/molbev/msab355 (PMC8788222; doi:10.1093/molbev/msab355)
Supplement: msab355_Supplementary_Data [file msab355_supplementary_data.zip › Supplementary Figures MBE revision - First look proof.pdf]

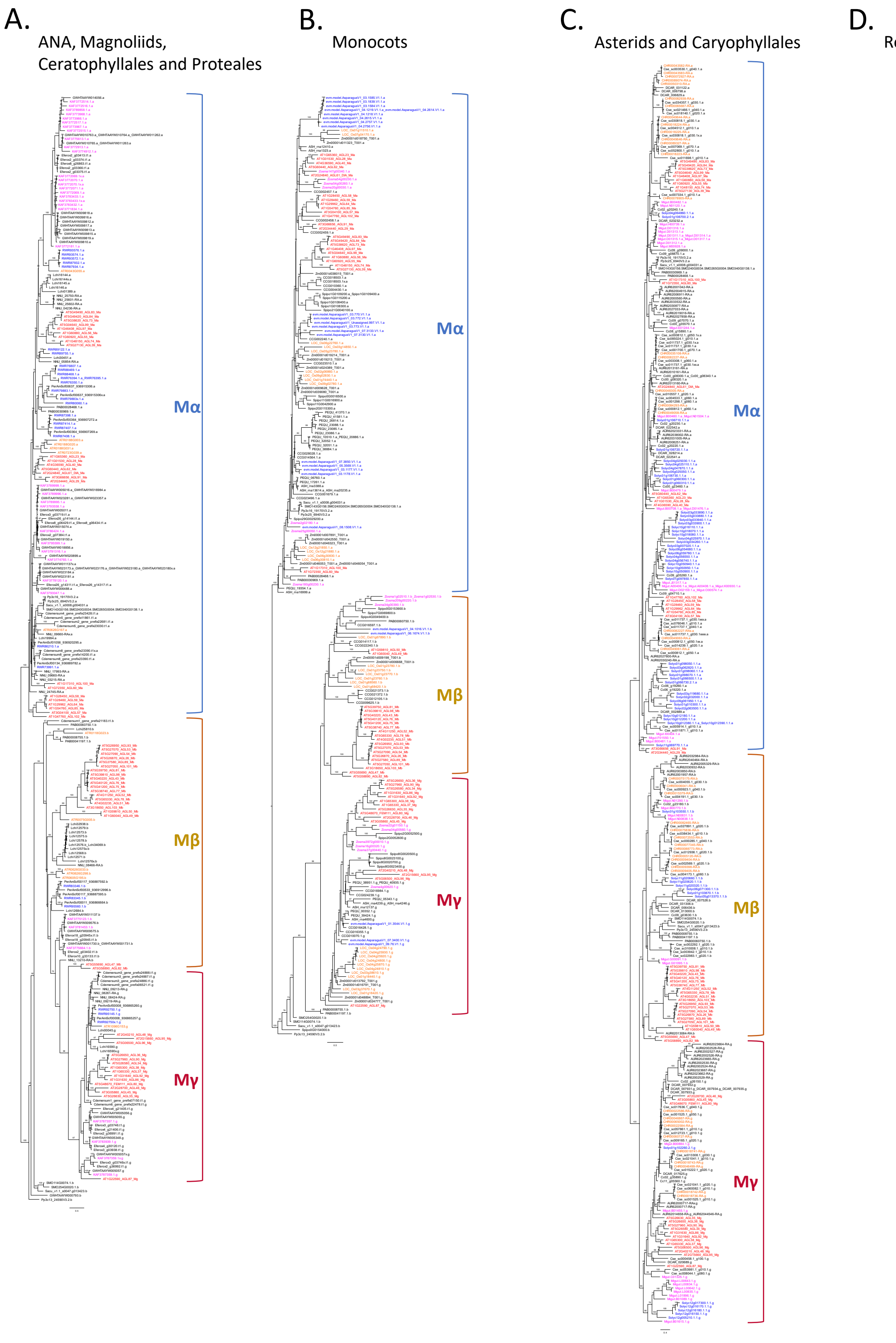

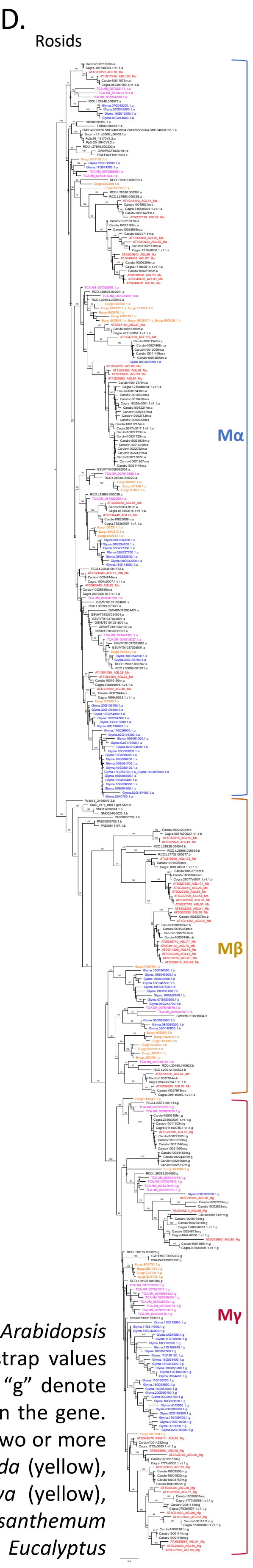

Fig. S1. Phylogeny of Type I MADS-box TFs in several focused groups of land plants with *Arabidopsis thaliana* MADS-box genes (highlighted in red) as references, shown by ML trees with bootstrap values supporting the branches. Gene identifiers in Supplementary Table S1-2. Suffixes of "a", "b", "g" denote Mα, Mβ or Mγ TFs, respectively; 'x' after gene identifier denotes the second MADS domain in the gene. Genes from selected species were colored to demonstrate the presence of Mγ genes and the two or more clusters of Mα genes. A. ANA, Magnoliids, Ceratophyllales and Proteales; *Amborella trichopoda* (yellow), *Nymphaea thermarum* (purple), *Cinnamomum camphora* (blue). B. Monocots; *Oryza sativa* (yellow), *Zostera marina* (purple), *Asparagus officinalis* (blue). C. Asterids and Caryophyllales; *Chrysanthemum nankingense* (yellow), *Erythranthe guttata* (purple), *Solanum lycopersicum* (blue). D. Rosids; *Eucalyptus grandis* (yellow), *Theobroma cacao* (purple), *Glycine max* (blue).

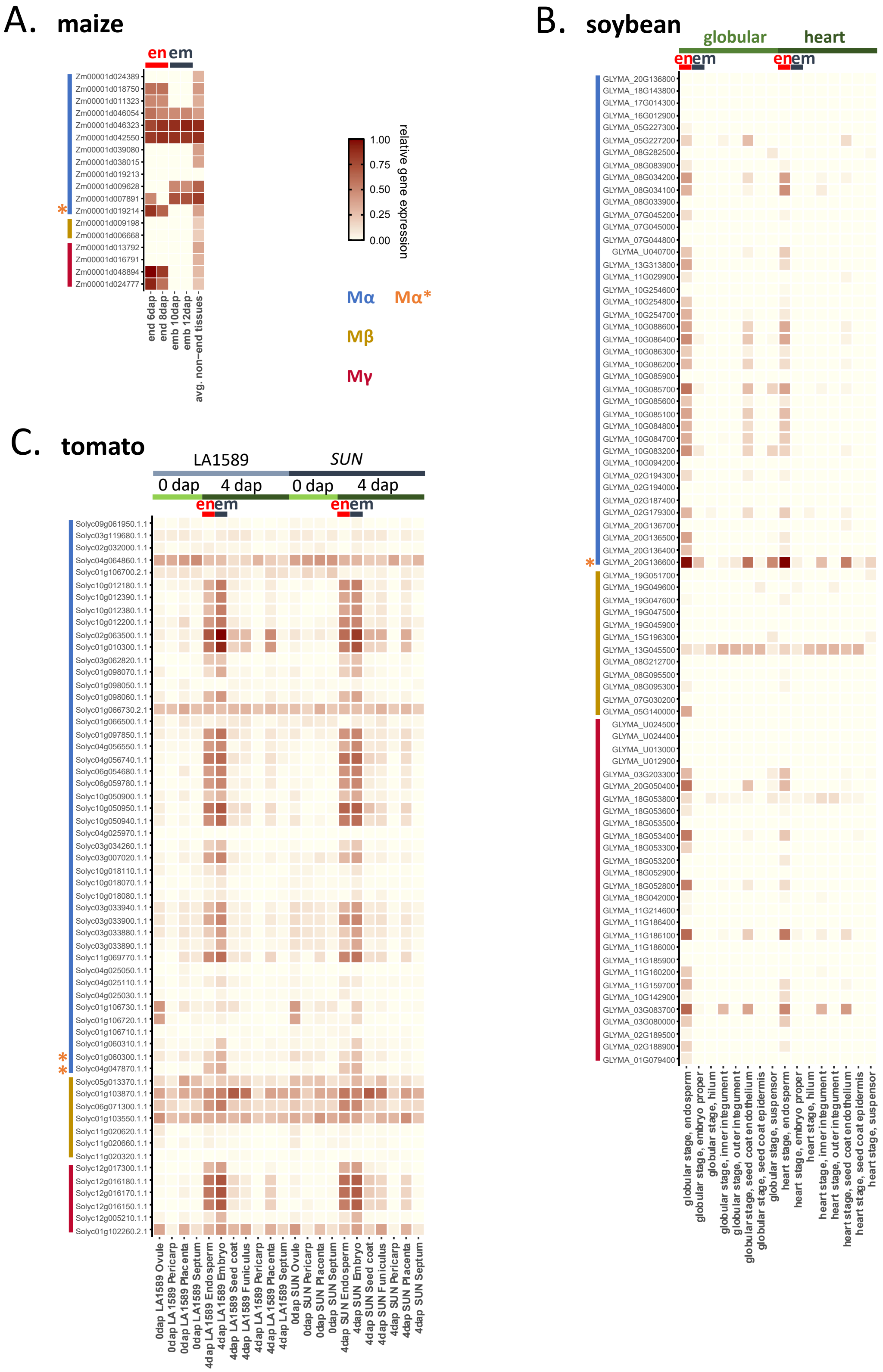

Fig. S2. Expression of Type I MADS-box genes in reproductive tissues of maize (A), soybean (B) and tomato (C), showing endosperm-expression of Mγ genes. The expression values were normalized into a 0-1 spectrum, with the max value set as 1. A. Gene expression levels at two developmental stages of seed tissues in maize and average expression level in non-endosperm tissues across the whole plant. B. Gene expression levels at two developmental stages of seed tissues in soybean. C. Gene expression levels in two genotypes (LA1589 / *SUN*) of seed and fruit tissues in tomato, before and after fertilization. en/end: endosperm; em/emb: embryo; dap: days after pollination.

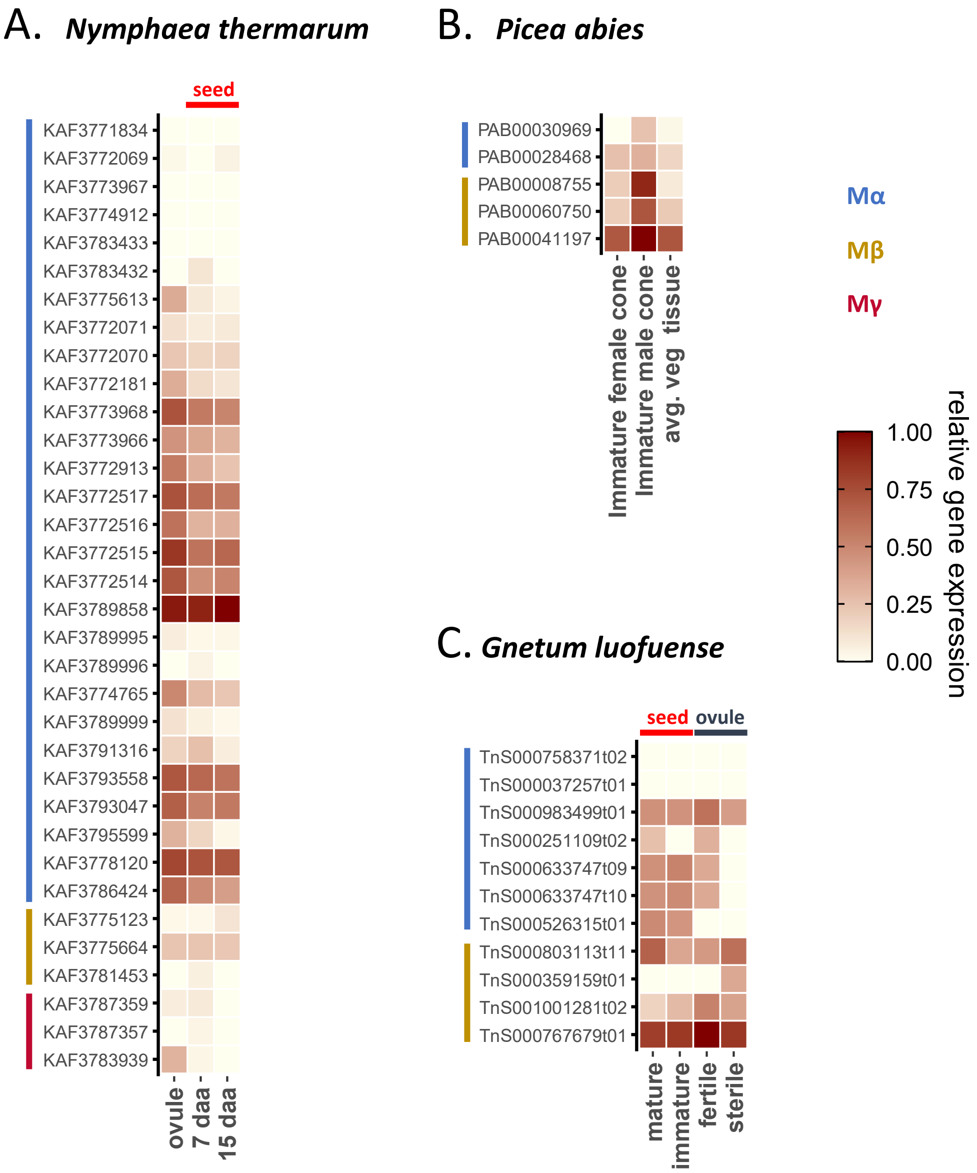

Fig. S3. Expression of Type I MADS-box genes in reproductive tissues of *Nymphaea thermarum* (A), *Picea abies* (B) and *Gnetum luofuense* (C), showing Mβ expression. The expression values were normalized into a 0-1 spectrum, with the max value set as 1. A. Gene expression levels at unfertilized ovule and two developmental stages of seeds in *Nymphaea thermarum*; daa: days after anthesis. B. Gene expression levels of female and male cones in *Picea abies* and average expression level in vegetative tissues; veg: vegetative. C. Gene expression levels at mature vs immature seeds in *Gnetum luofuense* and fertile vs sterile ovules.

A.

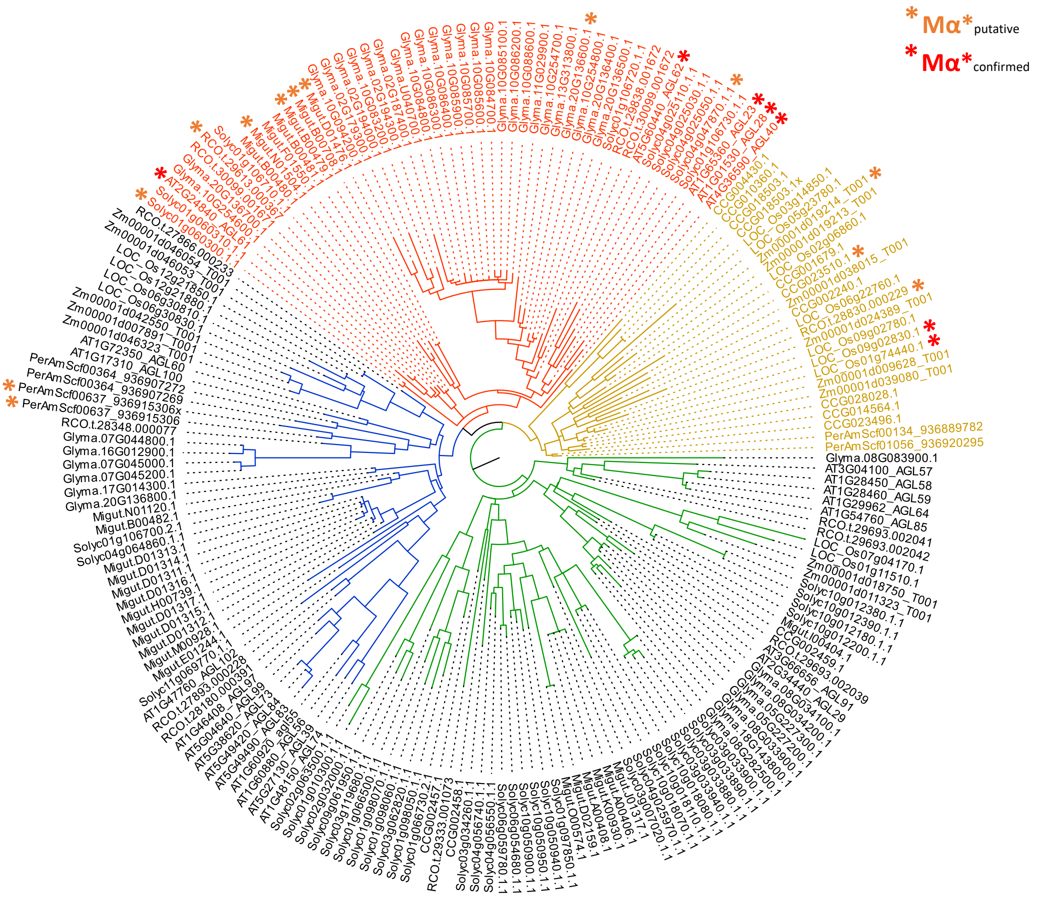

B.

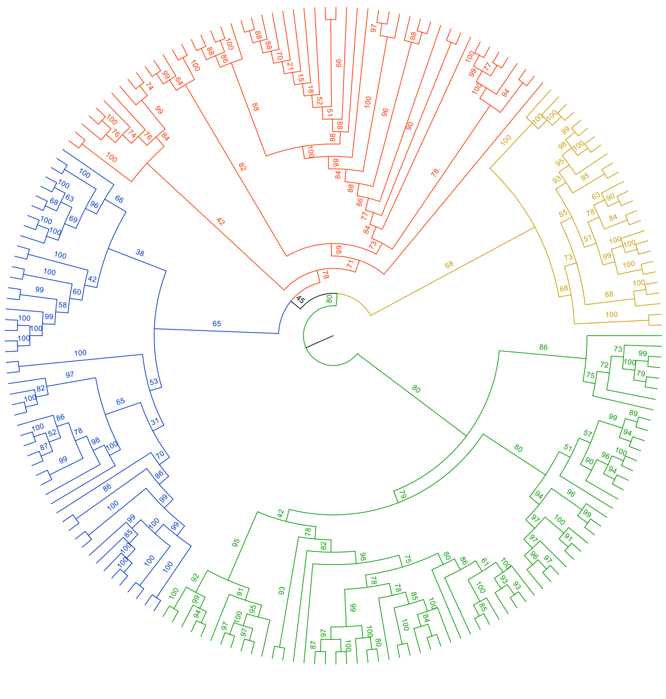

Fig. S4. The evolution of Mα* genes. A. Phylogeny of Mα genes from species with expression data from endosperm or seeds. The red clade has the confirmed Mα* genes in *Arabidopsis thaliana*, and the yellow clade has the confirmed Mα* genes in rice. Genes in the red and yellow clades are considered putative Mα* if they are expressed preferentially in the endosperm. The avocado genes in the red and yellow clades are weakly expressed (Fig.3), but the gene PerAmScf00637_936915306 (with two MADS domains annotated and shown in the phylogeny) in the sister clade was predicted as functional Mα*. B. A replicated tree of cladogram form labelled with the bootstrap support values for the branches. The internal clustering pattern within clades are better supported with values above 90. The basal branches connecting the major clades generally have supportive values below 80, suggesting that the backbone topology might not be fully resolved, and alternative topologies are equally possible, see Fig. S5.

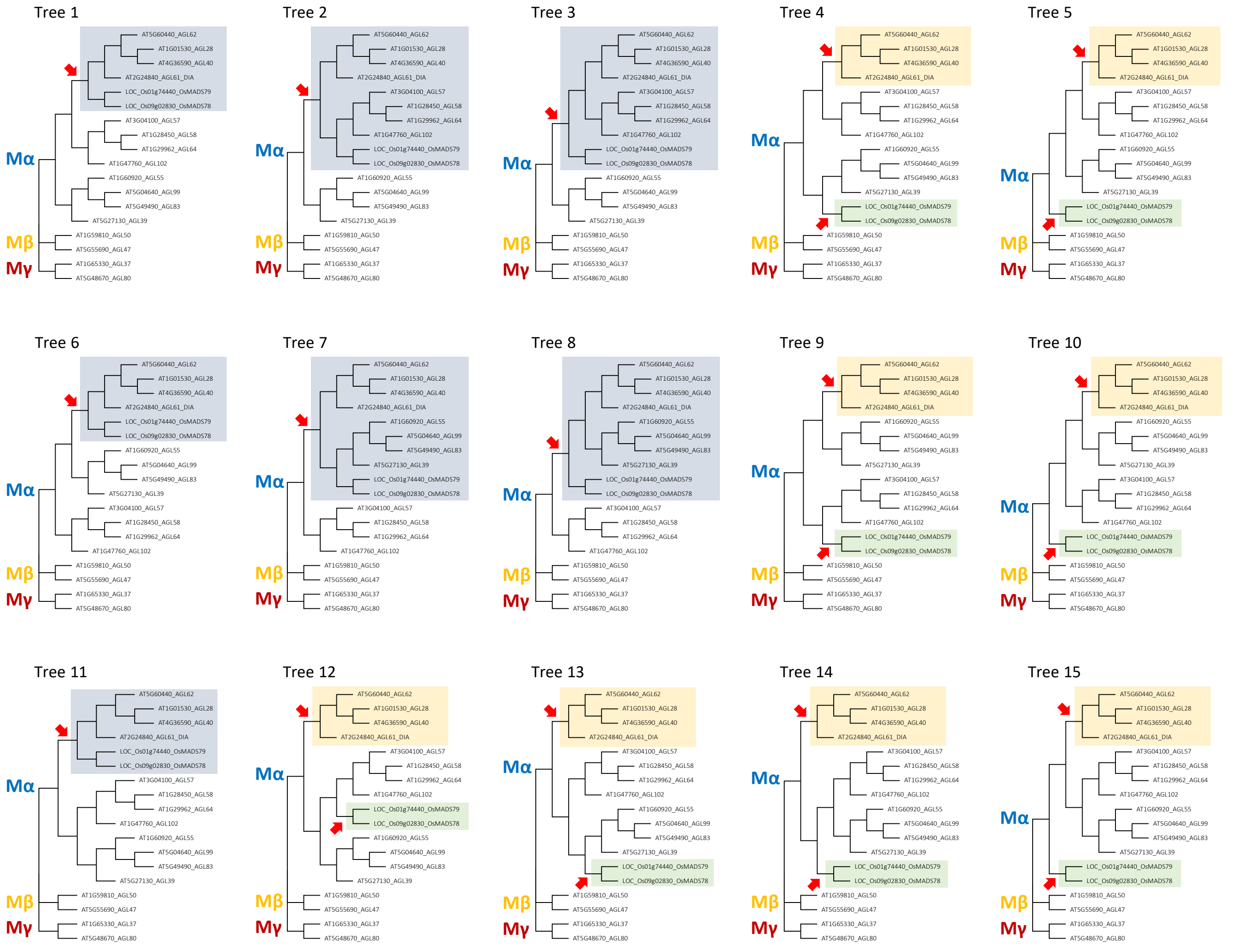

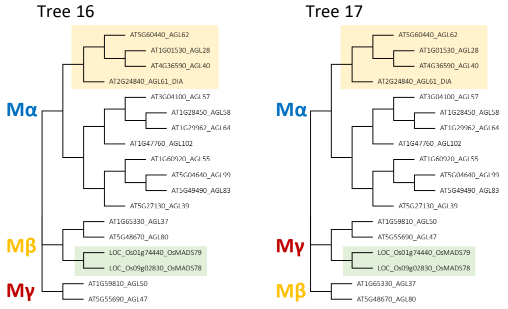

Fig. S5. Topology of different phylogenetic trees tested by IQ-tree representing two hypotheses of Mα* evolution. Amino acid sequences of MADS domains were aligned as input. In the constrained trees, Arabidopsis Mβ and Mγ were always used as outgroups. Three subclades of Arabidopsis Mα were shuffled due to uncertain relatedness. Rice Mα* were swamped to cluster with different Arabidopsis clades, illustrating two hypotheses: a shared origin of Mα* (blue clade) as in Trees 1-3, 6-8 and 11; independent rise of Mα* in Arabidopsis (pink clade) and in rice (green clade) as in Trees 4-5, 9-10 and 12-15. Red arrows point to the putative branches giving rise to Mα* clade. Neither hypothesis is rejected, nor is significantly better than the other. As negative controls, Trees 16 and 17, with rice Mα* forced to cluster with Arabidopsis Mβ or Mγ, are rejected. Stats of tree topology tests from IQ-tree are listed.

Tree	logL	deltaL	bp-RELL	p-KH	p-SH	p-WKH	p-WSH	c-ELW	p-AU
1	-2218.68	1.9379	0.0124 -	0.19 +	0.598 +	0.19 +	0.707 +	0.0374 +	0.259 +
2	-2218.86	2.1196	0.0015 -	0.152 +	0.561 +	0.151 +	0.723 +	0.0253 -	0.135 +
3	-2218.86	2.1196	0.0021 -	0.152 +	0.562 +	0.152 +	0.704 +	0.0253 +	0.131 +
4	-2218.88	2.1377	0.0027 -	0.146 +	0.561 +	0.146 +	0.678 +	0.0236 -	0.131 +
5	-2218.74	1.9911	0.0071 -	0.187 +	0.605 +	0.185 +	0.749 +	0.0318 +	0.184 +
6	-2218.54	1.7999	0.0434 +	0.219 +	0.613 +	0.219 +	0.724 +	0.0483 +	0.358 +
7	-2218.66	1.9127	0.0237 -	0.195 +	0.6 +	0.195 +	0.704 +	0.0377 +	0.147 +
8	-2218.24	1.4999	0.0831 +	0.277 +	0.671 +	0.276 +	0.771 +	0.0705 +	0.461 +
9	-2218.35	1.6059	0.0553 +	0.256 +	0.662 +	0.254 +	0.773 +	0.0596 +	0.276 +
10	-2218.35	1.6051	0.0678 +	0.256 +	0.663 +	0.254 +	0.814 +	0.0598 +	0.375 +
11	-2218.69	1.9503	0.0309 +	0.189 +	0.597 +	0.189 +	0.724 +	0.0361 +	0.189 +
12	-2216.75	0.010074	0.157 +	0.457 +	0.892 +	0.457 +	0.938 +	0.17 +	0.632 +
13	-2216.75	0.010094	0.211 +	0.456 +	0.892 +	0.456 +	0.937 +	0.17 +	0.629 +
14	-2216.74	0	0.277 +	0.543 +	1 +	0.543 +	0.915 +	0.173 +	0.69 +
15	-2218.72	1.9708	0.0243 +	0.184 +	0.608 +	0.184 +	0.758 +	0.0322 +	0.175 +
16	-2245.65	28.907	0.0001 -	0.0009 -	0.0016 -	0.0009 -	0.0027 -	1.19E-04 -	4.77E-05 -
17	-2245.65	28.907	0.0001 -	0.0009 -	0.0016 -	0.0009 -	0.003 -	1.19E-04 -	5.78E-05 -

deltaL : logL difference from the maximal logl in the set.
bp-RELL : bootstrap proportion using RELL method (Kishino et al. 1990).
p-KH : p-value of one sided Kishino-Hasegawa test (1989).
p-SH : p-value of Shimodaira-Hasegawa test (2000).
p-WKH : p-value of weighted KH test.
p-WSH : p-value of weighted SH test.
c-ELW : Expected Likelihood Weight (Strimmer & Rambaut 2002).
p-AU : p-value of approximately unbiased (AU) test (Shimodaira, 2002).

Plus signs denote the 95% confidence sets (possible topology).
Minus signs denote significant exclusion (rejected topology).
