# Supplementary material for: Endosperm Evolution by Duplicated and Neofunctionalized Type I MADS-Box Transcription Factors
Source: Mol Biol Evol. 2021 Dec 13;39(1):msab355. doi: 10.1093/molbev/msab355 (PMC8788222; doi:10.1093/molbev/msab355)
Supplement: msab355_Supplementary_Data [file msab355_supplementary_data.zip › MBE reply to referees.docx]

Response to reviewers’ comments

We thank both reviews for evaluating our manuscript and constructive comments for improvement. Our comments to the reviewers’ concern are marked in blue.

Reviewers' comments:

Reviewer: 1

Comments to the Author
MADS-box TFs play indispensable roles for plant development. To date, however, our understanding of the functions and their evolution of type-I MADS are still very limited. The manuscript entitled “Endosperm evolution by duplicated and neofunctionalized Type I MADS-box transcription factors” discussed the evolution and their importance for endosperm development of type I MADS-box TFs. By generating a comprehensive phylogenetic tree of type-I MADS and using previous published MADS interactome data, as well as the public available transcriptome data, the authors found that the angiosperm-specific Type I MADS-box clades of Mγ and Mγ-interacting Mα genes (Mα*) can be tracked back to the ancestor of all angiosperms. Mα* genes preferentially expressed in the endosperm and likely are essential for endosperm development by interacting with Mγ. The findings are interesting. However, because the main finding of the manuscript is conceptional, I have several concerns.

1.      Fig.1, ATR0118G023 is showed in wrong color. It is a Mγ MADS rather than a Mβ MADS. I assumed this gene is from Amborella, if it is true, the authors should highlight it as that did for ATR1096G153.

ATR0118G023 is a highly divergent gene, with an incomplete MADS domain that either appeared in the Mγ or Mβ lineage in different trees with a long branch and low confidence. In the tree shown in Fig. S1 it turned into the Mβ clade. We do not have confidence to conclude it as Mγ derived. Furthermore, it is incomplete and putatively non-functional, we therefore consider ATG1096G153 as the one and only Mγ in *Amborella*. The Fig. 1 is revised.

The Mβ MADS of Amborella should be indicated in this figure as well for better supporting the finding “at least one ancestral angiosperm Mβ gene and one ancestral angiosperm Mγ gene inherited in all the descendant lineages of angiosperms”.

Done as suggested.

2.      As showed in Fig.1, there are three Mβ MADS and three Mγ MADS that encoded by the maize genome. This result is conflict with the data showed in Fig.S2 and Fig.3, which showed there are two Mβ MADS and four Mγ MADS in maize. I did not compare the data showed in different figures for other species. The authors should double check the data for accuracy.

Thanks for pointing this out. The gene of concern, Zm00001d024777, has been rechecked and corrected. “Two Mβ MADS and four Mγ MADS in maize” is correct. The corresponding data between figures have been corrected.

3.      In Fig.3, I cannot see “the expression level of Mβ genes was SUBSTANTIALLY lower compared to the corresponding Mγ expression” in tomato.

We agree and removed the word “substantially”.

4.      There is another finding can be used to strengthen the authors’ hypothesis “the function of Mβ is dispensable in the context of endosperm regulation”. Cheng et al. (2020, Plant Journal; 2021, Plant Communications) found that in the defective endosperm that produced by the rice PRC2 mutants, many Mα and Mγ MADS genes were activated, whereas Mβ genes did not show such activation, indicating Mα and Mγ MADS are likely more important for endosperm development, at least for cellularization. This was also observed by Zhang et al. (2018) in Arabidopsis. Most majority of the type I MADS genes of the C2 group that activated in PRC2 mutants were Mα and Mγ.

Thanks for this suggestion. We are aware of these findings and also consider them in agreement with our findings. However, we decided not to complicate the manuscript by discussing role of PRC2 in regulating Type I MADS-box genes in the endosperm, which is tangent to this project.

5.      Given AGL62, and possibly AGL40, showed expression in vegetative tissues in Fig.2A, the statement “Genes encoding for the obligate Mγ-interacting Mα TFs (Mα* hereafter) were specifically expressed in reproductive tissues and co-expressed with PHE1/2 and other genes encoding for Mγ TFs in the endosperm” is not correct.

We agree and revised to “preferentially” instead of “specifically”.

In addition, please indicate which AGLs are PHE1/2 in the figures.

We put the information in the legends.

6.      It is ambiguous to me for defining a Mα* MADS. The authors identified a group of Mα MADS in the same phylogenetic branch, which showed abilities to interact with Mγ MADS, but not with Mβ MADS, in yeast. Therefore, they define them as the Mα* TFs. However, then the authors claimed that, bona fide, Mα* TF is expected to have central cell / endosperm-enriched or endosperm-specific expression and interacts with Mγ TFs. As showed in Fig.2, AGL64 displayed a similar expression pattern as that of Mα*, and it can also interact with AGL48, a Mγ MADS. Is AGL64 a Mα* MADS?

Since AGL64 can interact with Mβ (AGL77) based on Y2H data, it is not a bona fide Mα*. The dual interaction with both Mβ and Mγ is inferred as the ancestral “undifferentiated” status of Mα genes.

Next, aiming to support the idea that the Mα* TFs are essential for endosperm development, the authors cited Paul et al.’s work as another example in rice, which showed that OsMADS78 and 79 are required for seed development. Indeed, MADS78/79 are able to interact with MADS89 (a Mγ), however, in comparison to the other Mα TFs of Arabidopsis, MADS78/79 showed much further phylogenetic distances to the Arabidopsis Mα* TFs (Fig S1.B). In addition, it is still unknown whether MADS78 and 79 have the ability to interact with Mβ MADS, and whether they exclusively expressed in the endosperm. Therefore, I suspected that the Mα* group is evolved and subfunctionalized after gene duplication in Arabidopsis, there possibly is no so-called Mα* existed in rice.

We define an Mα* TF by the function, not dependent on the origin. Based on the transcriptomic data from Sakai et al. (2011) and Davidson et al. (2012), OsMADS78/79 are barely expressed in vegetative tissues. Thus, they are preferentially expressed in the endosperm, the same as the Mα* in Arabidopsis.

It turned out to be difficult to resolve the phylogeny of Mα TFs, and we did AU tests as suggested by Reviewer 2, which did not reject the hypothesis that OsMADS78/79 and Arabidopsis Mα* TFs derived from the same ancestral putative Mα*, while this is not the only possible explanation. So, we also present an alternative hypothesis of independent specialization events of Mα* in different lineages.

In addition, indicate MADS78 and 79 after their locus IDs in the related figures.

We added the information in the legends.


Reviewer: 2

Comments to the Author
Qiu and Köhler analyze sequence, gene expression and yeast-2-hybrid data to extend make a convincing case for the role of Mγ and Mα* heterodimers in the evolution and development of the angiosperm endosperm.  The manuscript extends a recently published functional analysis of Mγ and Mα genes in rice (Paul et al. 2020 Plant Phys.) to diverse flowing plant lineages including non-grass monocots, eudicots and magnoliids.  Further, the well-executed comparative analyses illustrate the power of phylogenically informed comparative analyses using available gene expression and functional data.

I have just a few suggestions for the authors’ consideration:

As the authors acknowledge, phylogenetic inference of MADS box gene relationships is difficult given their relatively short length and conserve amino acid sequences.  Nonetheless, it may be possible to test for the monophyly of Mα* genes and the significance of discordance between the trees shown in Figures 1 and 2C in the main text.  At the very least, “putative” and “confirmed” (as specified in Fig 3) Mα* genes should be indicated in Figures 1 and S1.

Due to the large amount of Type I MADS-box genes in tomato, soybean and monkeyflower, we did not include them into Fig. 1. Furthermore, both Fig. 1 and Fig. S1 include genes from many of species without expression data; we therefore felt it misleading to label Mα* in these figures as we cannot surely rule out that other species do not have Mα*. We added Fig. S4 showing the phylogeny of all genes from species with expression data, and labelled confirmed and putative Mα*.

Even if there was a single origin of Mα* gene function (as asserted in Fig 4), is not surprising for Mα* genes to be resolved as polyphyletic with poor/mediocre support in the complex gene trees presented in Figures 1 and S1, but the authors can perform an SH or AU test to assess whether the data allows for rejection monophyly.  The authors could also discuss alternative explanations for polyphyly or paraphyly of Mα* genes, i.e. multiple origins or duplication and shift in function.

As suggested, we performed AU tests and neither the monophyly nor paraphyly of Mα* is rejected. We have extended the alternative hypothesis previously mentioned, and present both scenarios of Mα* evolution with more details.

In addition, I encourage the authors to replace the phrases “earliest-diverging”, and “early-diverging” :
•       P3, L15 – “…sister to all other angiosperms…”
•       P6, L27 – “…representing the successive sister lineages to all other angiosperms…”
•       P7, L7 – “…ancestral land plants…” or “…earliest land plants…”

We rephrased as suggested.

VERY minor suggestions:

P5, L27 – change “Type II TFs can homodimerize, and usually heterodimerize…” to “Some Type II MADS-box TFs can homodimerize, but many typically heterodimerize…

We rephrased as suggested.

P5, L 30-32 – Why does the phylogenetic relatedness of Mβ and Mγ genes imply that they would barely interact?

We added the explanation in the text.

P7, L52 – In addition to citing CoGe’s url, the publication describing CoGe workflows could be cited - https://doi.org/10.1002/pld3.8

We added this citation and the corresponding reference.
